# Evaluating results of the implementation research MOOC using Kirkpatrick’s four-level model: a cross-sectional mixed-methods study

**DOI:** 10.1136/bmjopen-2021-054719

**Published:** 2022-05-03

**Authors:** Bella Ross, Michael J Penkunas, Dermot Maher, Edith Certain, Pascal Launois

**Affiliations:** 1 Monash College, Docklands, Victoria, Australia; 2 United Nations University International Institute for Global Health (UNU-IIGH), Cheras, Kuala Lumpur, Malaysia; 3 Special Programme for Research and Training in Tropical Diseases (TDR), WHO, Geneva, Switzerland; 4 Consultant - Special Programme for Research and Training in Tropical Diseases (TDR), WHO, Geneva, Switzerland

**Keywords:** public health, international health services, medical education & training

## Abstract

**Introduction:**

An implementation research (IR) massive open online course (MOOC) was developed by the Special Programme for Research and Training in Tropical Diseases, to address the scarcity of training in low-income and middle-income countries in the field of IR. The Kirkpatrick model was used to evaluate the IR MOOC as it is widely applied for evaluation of training and educational programmes. The Kirkpatrick model evaluates training programmes on four levels: reaction, learning, behaviour and results. This paper addresses the impact of the IR MOOC on participants’ professional practice.

**Methods:**

Findings are based on analysis of survey and interview data collected 1.5–2 years after the conclusion of the two 2018 IR MOOC offerings. Of the 3858 MOC participants, 748 responded to the anonymous online survey and seven of these respondents were interviewed. All data are self-reported.

**Results:**

The IR MOOC was successful in enhancing the professional practice of participants and for their organisations. Over 40% reported modifying or implementing changes in their professional work. Respondents reported that participation in the MOOC had improved their ability to conduct IR, enhanced their professional profiles and increased their opportunities for collaboration, research and job promotion. Respondents stated that the MOOC had improved their work quality and productivity, and allowed them to contribute to research, initiate and develop professional collaborations and train others in IR. Respondents reported an increase in applying for grants and scholarships and presenting and publishing work on IR after participating in the MOOC. Barriers applying the knowledge gained from the IR MOOC were experienced, for example, due to a lack of funding and lack of support from colleagues, managers and organisations.

**Conclusion:**

Participants perceived that the IR MOOC was successful in its aims of delivering medium-term and long-term results in relation to their own and their organisations’ professional outcomes.

**Strengths and limitations of this study**

- Past studies of the implementation research massive open online course (MOOC) for investigators in low-income and middle-income countries (LMICs) revealed considerable increases in learners’ implementation research knowledge as well as positive changes to their professional behaviour.
- This capstone evaluation demonstrated that the MOOC was successful in enhancing learners’ professional practice, both individually and for their organisations.
- We found that the MOOC had improved participants’ ability to conduct implementation research, elevated their professional profiles and improved their work quality and productivity.
- Future training can build off the success of the MOOC and use this resource to complement other training tools that strengthen the skills of both individuals and research teams working in LMICs.

## Background

The past two decades have seen an increasing recognition of the potential implementation research (IR) in improving the effectiveness of health interventions, particularly in low-income and middle-income countries (LMICs).^1^ IR intends to identify implementation bottlenecks and inform strategies designed to overcome these barriers for efficacious health interventions that are implemented in real-world settings and scaled beyond highly controlled clinical trials.^2 3^ The maturing of IR as a scientific field and its growing popularity has inspired a cadre of implementation scientists across disciplines^4 5^ and geographies.^1 6^

IR is gaining ground within LMICs in particular, where implementation challenges are often encountered but inappropriately studied.^6 7^ The IR massive open online course (MOOC) was designed to address this gap by providing IR training to health professionals and students, particularly those living and working in LMICs. Because each implementation bottleneck is caused by context-specific challenges, local researchers who possess an in-depth knowledge of the community, health system and infrastructure are inherently best positioned to study implementation challenges and design methods to overcome these bottlenecks. A number of high-quality courses, training programmes and workshops in IR have been created in recent years, but the vast majority are out of financial or practical reach of learners in LMICs. The Special Programme for Research and Training in Tropical Diseases (TDR), cosponsored by the United Nations Children’s Fund, the United Nations Development Programme, the World Bank and the WHO, maintains perhaps the most complete and versatile library of training material*s* for burgeoning IR investigators within LMICs. This suite of training tools includes an open access IR toolkit, principles of IR course, a guide for publishing IR results, a specialised module on ethics in IR, and its flagship MOOC, which is being evaluated here.

### The IR MOOC

The MOOC examined here was developed by TDR to address the relative lack of IR training opportunities for investigators in LMICs. The five-module course focuses on applying IR concepts to increase the ‘real-world’ effectiveness of interventions for infectious diseases of poverty, such as malaria and neglected tropical diseases. MOOC content, along with interactive quizzes and discussion forums, is delivered over 6 weeks and uses worked case examples to illustrate key IR concepts. The MOOC is intended for researchers and health practitioners, with the goal of building knowledge of IR methods, purpose, and approaches among course participants.^8 9^ At the time of evaluation, the IR MOOC was available in English, French and Spanish with Russian, Chinese and Arabic versions under development. Additional modules for the MOOC are currently being developed by TDR and partners, and include specialised modules on qualitative methods, community engagement, and integrating gender and intersectionality into IR. Furthermore, the MOOC, which originally focused on infectious diseases of poverty, has been adapted for IR capacity strengthening around non-communicable diseases.^10^

Prior studies of the IR MOOC have demonstrated considerable increases in learners’ IR knowledge and positive changes to their professional behaviour.^11^ It has also been shown that the MOOC is reaching its intended audience of public health and research professionals in LMICs.^11^ Lessons learnt since the introduction of the MOOC include the importance of delivering the course in languages other than English, presenting geographically relevant case examples, and training course facilitators on stimulating discussion through the online forum.^12 13^

The current study builds on these findings to examine results, that is, if and how participants’ learning from the IR MOOC translated into their job outcomes and throughout their organisation, which is considered the ultimate impact of the training and in effect evaluates whether the training achieved its aims.

### Evaluating results using Kirkpatrick’s model

This study evaluates the IR MOOC using the Kirkpatrick Model.^14^ The model has been widely used to evaluate courses for learners in high-income settings,^15–19^ and is being applied to MOOCs targeting LMICs participants.^20^ The Kirkpatrick Model includes four levels of evaluation: (1) reaction, (2) learning, (3) behaviour and (4) results. This paper presents an evaluation of results, where results refers to improvement of job and organisational performance. The findings related to participants’ reaction^13^ and behaviour^11^ have been reported previously.

## Methods

### Survey data

The composition and methodology for the online survey have been described previously.^11^ In short, quantitative and qualitative data were collected between November 2019 and February 2020 through an online anonymous survey consisting of 43 open-ended, multiple choice, and 5-point Likert-type questions (ranging from ‘to a very small extent’ to ‘to a very large extent’). The survey captured participants’ demographics, satisfaction, change in behaviour, and results from the MOOC that impacted their professional work or that of their organisation. The survey, programmed in Qualtrics software,^21^ was sent via email in November 2019 to all participants (N=3858) from the two 2018 MOOC cohorts. Our aim was to include a diversity of perspectives among all who participated in the IR MOOC, not only those who earned the certificate of completion. A response rate of 19.4% (N=748) was achieved, with a 3% margin of error. Descriptive statistics for the quantitative data were calculated using both Qualtrics and Microsoft Excel.^22^ Data from the open-ended questions were thematically analysed following Braun and Clarke’s^23^ six phases: (1) familiarising oneself with the data, (2) generating initial codes, (3) searching for themes, (4) reviewing themes, (5) defining and naming themes, and (6) producing the report. The thematic coding scheme was produced through discussions between author 1 and the primary coder. Author 1 double-coded approximately 10% of the data to check for interrater reliability. Levels of agreement were greater than 90% and inconsistencies were resolved through discussion between the two coders. The reporting of this evaluation is informed by the CRe-DEPTH guidelines.^24^

### Interview data

On completion of the survey, respondents were asked to provide their contact details if they were interested in being interviewed regarding their experiences in participating in the MOOC. Out of 361 who volunteered, a convenience sample of 32 respondents were then contacted via email by author 1 to schedule a teleinterview. Potential interviewees were chosen in order to represent the nationalities, professions, genders and education levels of the MOOC participants. Of those invited, seven responded. They were provided with a plain language statement on the aims of the research project and participants provided written informed consent. Semistructured teleinterviews were conducted in June and July 2020. Nineteen questions built on the survey questions and focused on participants’ perceptions of the MOOC, their learning and ways in which the MOOC had impacted on their professional behaviour and their organisations. The semistructured teleinterviews ranged in duration from 22 to 50 min. The teleinterviews were audiorecorded and transcribed. Where relevant, the interview data is used to complement the findings from the survey data.

### Public involvement

The public was not involved in the design, conduct, reporting or dissemination plans for this research.

## Findings

### Demographics of MOOC registrants and survey and interview participants

**Figure 1:**
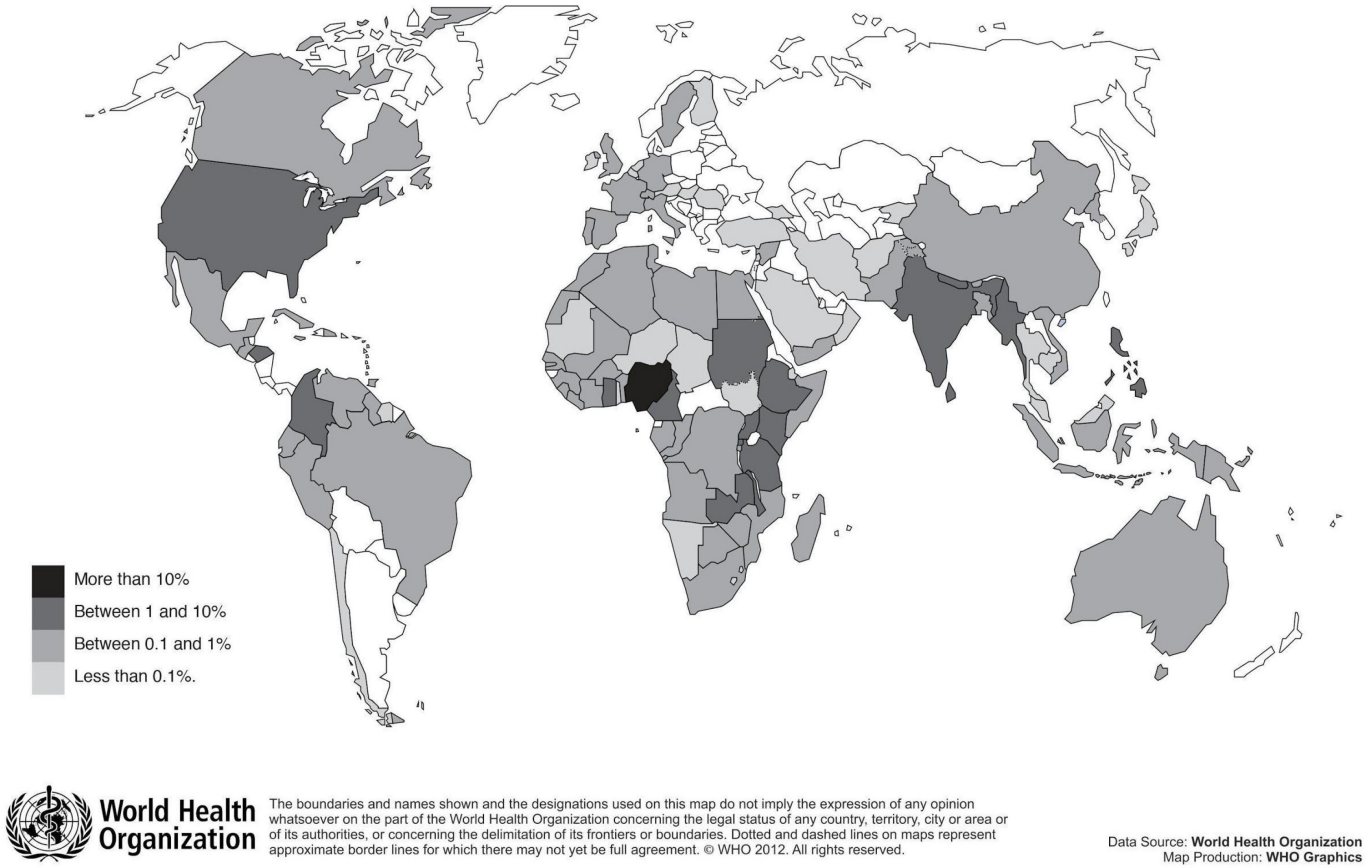
The most represented countries of MOOC registrants. MOOC, massive open online course.

As described previously,^11^ 3858 registrants participated in the May and October MOOC. Of these, approximately 30% (1163/3858) earned a certificate of completion. Registrants represented 115 different countries, the majority of whom (62.4%) came from the WHO African Region. Participants from the WHO South-East Asian Region accounted for 17.7%, the WHO Americas Region accounted for 9.9%, the WHO Eastern Mediterranean Region accounted for 5.5%, the WHO European Region accounted for 2.8% and participants from the WHO Western Pacific Region accounted for 2.2% Figure 1 displays the countries represented by the MOOC participants.

Table 1 provides a summary and comparison of the demographics of the MOOC participants and the survey respondents (also reported in Launois *et al*^11^). Overall, the survey respondents are largely representative of the MOOC participants.

**Table 1:** Demographic information of IR MOOC participants and survey respondents

	MOOC participants	Survey respondents
Sex	Female: 44% Male: 57%	Female: 44.1% Male: 55.9%
Age	Between 20–40 years: 77.5%	Between 26–40 years: 67.5%
WHO region	African Region: 62.4% South-East Asian Region: 17.7% Americas Region: 9.9% Eastern Mediterranean Region: 5.5% European Region: 2.8% Western Pacific Region: 2.2%	African Region: 69.4% South-East Asian Region: 12.6% Americas Region: 9.6% Eastern Mediterranean Region: 1.6% European Region: 4.1% Western Pacific Region: 2.7%
Profession	Public health researchers: 45% Public health officers: 15.5% General practitioners: 11.1% Students: 11%	Public health researchers: 31.2% Public health officers 17.4% Students: 15.3% Teachers: 11.4% General practitioners: 9.3%
Education level attained	Master’s degree: 41.5% Bachelor degree: 24.7% PhD/Doctorate: 12.6%	Master’s degree: 57.1% Bachelor degree: 25.6% PhD/Doctorate: 17.3%
Certificate of completion obtained	Of the total initially enrolled: 30.15% Of those who completed the course: 89.2%	70.6%

IR, implementation research; MOOC, massive open online course.

Table 2 presents the demographics of the seven interviewees. Interviewees were made up of five males and two females. One interviewee held a bachelor’s degree and three each held a master’s and doctoral degree. Four of the interviewees’ age range was 31–35, one was aged between 26 and 30 and two were aged between 46 and 50. Although a roughly equal number of male and female respondents were invited to be interviewed (32 in total: female=43.75%, male=56.25%), note that mainly males responded. Six out of seven participants are living and working in Africa and one is living and working in Germany. Regarding profession, three participants work as university academics. One participant is enrolled in a master’s degree in public health. Two participants work for non-governmental organisations, and are involved in public health projects. One participant is a specialist doctor and one is a clinical researcher. In terms of research experience, four participants have been involved in public health research. Two participants have experience in clinical research. One participant’s research interest is in capacity building and policy-making.

**Table 2:** Demographic information of interviewees. Participants’ secondary country of residence is presented in brackets.

	Country of residence	Duration
Int 1	Nigeria	45 min
Int 2	Ghana/(Mali)	28 min
Int 3	Sudan/(Lebanon)	47 min
Int 4	Egypt	22 min
Int 5	Rwanda	30 min
Int 6	Nigeria	50 min
Int 7	Germany	23 min

From the survey, the most commonly cited reasons for participating in the IR MOOC are provided in table 3.

**Table 3:** Survey respondents’ most commonly cited reasons for participating in the MOOC

Reason for participation in the MOOC	Percentage
To gain knowledge and understanding of IR	86.8%, F=86.6%, M=86.9%
To apply the knowledge and tools in research	63.4%, F=62.2%, M=64.4%
To apply the knowledge and tools in practice	55.2%, F=54.1%, M=56%
For self-learning purposes	47.6%, F=49.1%, M=46.4%
To further specialise in their field	36%, F=36.3%, M=35.8%
To obtain a certificate of completion	30.3%, F=27.5%, M=32.6%

MOOC, massive open online course.

All of the interviewees likewise cited self-motivated reasons for participating in the MOOC. The main reason was that the course was relevant to their job, research interests or educational background. They received information about the course from sources other than their workplace.

### Results from participating in the MOOC

As a result of the IR MOOC, survey respondents had undertaken a range of professional activities, as shown in table 4. Over one-third had modified or implemented changes in their professional practice and initiated or conducted research, while a quarter had initiated or developed new professional collaborations.

**Table 4:** Survey respondents’ most commonly cited professional activities undertaken as a result of participating in the MOOC

Professional activity	Percentage
Modified or implemented changes in their professional practice	41.7%, F=35.3%, M=46.7%
Initiated or conducted research	35.9%, F=34.7%, M=36.8%
Initiated or developed new professional collaborations	24.4%, F=23.8%, M=24.9%
Developed or submitted publications	16%, F=16.6%, M=15.6%
Applied for or been awarded grants	14.6%, F=12.2%, M=16.5%

MOOC, massive open online course.

These findings were reflected in the interview data, with four participants reporting that they improved how they conducted their research:

I think for me one of the benefits of taking the MOOC was to kind of formalise the way that we approach some of our research more within that framework, to make it more objective and outcome oriented. (Interviewee 7)

Three of the interviewed participants stated that they have improved the way they conduct research and solve problems as a result of participating in the MOOC:

It [the MOOC] helped me, I think mainly to understand the—to help me to solve one of our other thing—one of our study in Mali and this study was facing a lot of challenge in community and there was fake news, that sort of thing. So MOOC helped me very much to overcome this challenge. (Interviewee 2)

A majority of surveyed respondents (80.1%) had passed on knowledge gained from the MOOC to colleagues or peers. Four of the interviewees likewise supported this, stating that they had either shared their knowledge with their teams or advised their teams on IR projects:

I actually shared some of the information with the rest of my team afterwards. (Interviewee 7)

Another participant perceived that passing on knowledge of IR to colleagues was necessary for further collaboration and career prospects in this area:

There’s a gap in the knowledge because not all of us are really familiar with implementation research so—and those of us who have a good knowledge of it, or let’s say a basic knowledge — we try as much as possible to teach other people, train them on implementation research, so that we can work together as a team and achieve our main goal. So for career paths, it’s a big challenge because it’s just starting and we don’t know where it will end up, but it’s always good to start so that you know where you are going to. (Interviewee 1)

### Opportunities for career enhancement

In relation to whether participation in the MOOC had increased survey respondents’ professional opportunities for promotion, just under half (52.4%, F=48.4%, M=55.5%) felt it had while just under half (47.6%, F=51.6%, M=44.5%) disagreed. Of the 218 respondents who elaborated through the open-ended questions, almost one quarter cited improved research performance and just over ten percent cited an enhanced professional profile as contributing factors to possible career enhancement. Many stated that they felt more confident professionally due to an increase in IR knowledge, that they felt able to conduct and contribute to research in the field of IR, and that the MOOC had opened doors for them professionally. The most common themes given in response to these questions are:

- Improved research performance (N=52).
- Enhanced professional profile, professional advantage, recognition, awards and publications (N=25).
- Opportunity for collaboration and research (N=23).
- Job promotion (N=21).
- Success or confidence in applying for or retaining a job (N=20).
- Research grants (N=16).

#### Improved research performance

Responses to the open-ended survey questions indicated that the MOOC had improved participants’ research performance, which played a key role in their career enhancement opportunities. This includes writing research proposals, participating in conferences, addressing challenges in research projects, and contributing to research teamwork in general. One respondent stated:

I debate using examples of existing research, discuss from an informed point of view and I continue to lobby for stakeholder involvement in decision making at all levels. (Survey respondent, Public health researcher, Uganda, Obtained certificate)

#### Enhanced professional profile, professional advantage, recognition, awards and publications

The MOOC contributed to respondents’ professional profiles in a range of ways, including recognition from peers, awards and publications. Statements include:

I received an award for best abstract [conference name] 2019 in Mexico. (Survey respondent, Public health researcher, Zimbabwe, Obtained certificate)

As a result of the knowledge I gained from the MOOC, I have been able to publish two papers which led to my recent promotion to my current position. (Survey respondent, Public health researcher, Nigeria, Obtained certificate)

#### Opportunity for collaboration and research

Participants felt that the MOOC had provided them with increased opportunities for research and collaboration with others:

I am currently discussing with my institute directors to make IR as an additional research unit to our existing structure. (Survey respondent, Public health researcher, Papua New Guinea, Obtained certificate)

#### Job promotion

Many respondents stated that the MOOC had directly assisted them in achieving job promotions, which included both promotion to a higher position or an increase in salary. One respondent stated:

I am now part of a national chapter that is training and creating awareness of implementation research. (Survey respondent, Teacher, Rwanda, Obtained certificate)

#### Success or confidence in applying for or retaining a job

A number of respondents felt that the MOOC had provided them with either success or confidence in applying for or retaining a job. For example, one respondent stated:

My contract was renewed for an extra year thanks to the skills I implemented in my day-to-day management of the project, which was very satisfactory. (Survey respondent, Public health officer, Cameroon, Obtained certificate)

#### Research grants

Respondents were successful in attracting research grants as a result of participation in the MOOC, as illustrated by the following statement:

I got a grant which I am going to use to design an implementation study. (Survey respondent, Researcher, Uganda, Obtained certificate)

#### Benefits to place of employment

The majority of survey respondents (66.3%, F=62.1%, M=69.6%) felt that their participation in the MOOC had benefited their place of employment with 276 respondents elaborating on how this was the case. Benefits included improvements in participants’ work, increases in participants’ IR knowledge and evaluation skills, and that participants shared the knowledge they gained from the MOOC with others within their organisation. These results were ultimately beneficial and resulted in better outcomes for participants’ organisations. The following outlines the themes of these responses.

- Improved work quality, efficiency or productivity (N=94).
- Contribution to research activities and collaboration (N=35).
- Teaching, training, supervising, mentoring or advising students or colleagues (N=33).
- Application of the knowledge and skills gained (N=29).
- Enhanced knowledge (N=24).

#### Improved work quality, efficiency or productivity

Respondents cited improvements in work quality, efficiency and productivity. These improvements were found at both the individual and institutional level. The following statement reveals one respondent’s perception:

Better research questions have been developed and implemented which has led to improvement of quality of services we provide. (Survey respondent, Public health officer, Uganda, Obtained certificate)

#### Contribution to research activities and collaboration

Participation in the MOOC contributed to respondents’ research activities and collaboration, including establishing or expanding collaboration and partnerships at their place of work. Representative statements include:

It has enabled extension of new professional collaboration research partners and nations especially in the field of IR to our institute. (Survey respondent, Public health researcher, Papua New Guinea, Obtained certificate)

Yes, the training has increased my opportunities. I am part of a research team that we have set up a kind of NGO for Health-Environment and Climate Change. As a result, I bring a lot of research opportunities in tenders. I also contribute to the development of projects looking for funding. (Survey respondent, Teacher, Côte d'Ivoire, Obtained certificate)

#### Teaching, training, supervising, mentoring or advising students or colleagues

A benefit of the MOOC cited by respondents concerned their improved abilities in teaching, training, supervising, mentoring or advising students or colleagues, as illustrated by the following quotes:

I am able to advise on ways to engage all stakeholders including beneficiaries. (Survey respondent, Student, Malawi, Obtained certificate)

I am mentoring junior colleagues in my place of work and together we will increase the productivity and efficiency. (Survey respondent, Public health researcher, Nigeria, Obtained certificate)

#### Application of knowledge and skills gained

Respondents stated they were able to apply the knowledge and skills gained from the MOOC in their professional work. One survey respondent applied the knowledge daily:

I apply the knowledge gained from the course on a daily basis. (Survey respondent, Administrator, Ghana, Obtained certificate)

An interviewee outlined how the MOOC had assisted them in their work:

So it [the IR MOOC] has really, really helped me. Right now I can identify how to assess interventions, how to define the implementation problem, how to design implementation challenges, and also test implementation strategies. (Interviewee 1)

### Barriers applying IR knowledge

Over 40% of survey respondents (41.5%, F=37.6%, M=44.4%) stated they had faced challenges or barriers to applying the knowledge gained from the MOOC in their professional work or research. Of those surveyed, 217 participants elaborated on their response. The most commonly cited themes are as follows:

- Lack of funding, finance, time and other resources (N=59).
- Lack of knowledge or interest from colleagues, teammates and peers (N=38).
- Lack of support from colleagues, managers, supervisors, organisations, institutions, or workplaces (N=24).
- Limited or no opportunities to apply IR (N=24).

#### Lack of funding, finance, time and other resources

The most commonly cited barrier was a lack of funding, finance, time and other resources, as illustrated in the following:

Funding and getting buy in from policy makers is usually not easy. (Survey respondent, Public health researcher, Nigeria, Obtained certificate)

#### Lack of knowledge or interest from colleagues, teammates and peers

Respondents cited lack of knowledge or interest from colleagues, teammates and peers as a common barrier to applying the knowledge and skills from the MOOC in their work:

Few people really know about this research, so if you explain others do not understand. (Survey respondent, Public health researcher, Tanzania, Obtained certificate)

An interviewed participant stated that a barrier to applying IR, in addition to limited opportunities for collaboration and mentorship, was due to a lack of training opportunities:

Also training opportunities—where there would be a kind of hands-on experience. Hands-on experience. Because like I said earlier, in our countries, we have problems of training and retraining. Our research requires training and retraining. It requires application directly in the field. Not on papers or in the classrooms. So that is actually what we are facing. (Interviewee 6)

#### Lack of support from colleagues, managers, supervisors, organisations, institutions or workplaces

Respondents felt there was a lack of support to use the knowledge and skills of IR gained. This lack of support was often discussed in terms of resistance and in connection with a lack of knowledge from colleagues, managers, supervisors, organisations, institutions, or workplaces. One surveyed respondent stated:

Knowledge of IR in Uganda is still low, so application of IR concepts faces resistance from other stakeholders. (Survey respondent, Public health researcher, Uganda, Obtained certificate)

These barriers were also found in relation to collaboration and teamwork:

Sometimes it is difficult to collaborate with other people in research in developing IR proposals. (Survey respondent, Public health researcher, Cameroon, Obtained certificate)

#### Limited or no opportunities to apply IR

Respondents stated they had limited or no opportunities to apply IR in their work due to, for example, the limitations of their current professional positions, as illustrated in the following statement:

I did not yet have an opportunity/a project to apply the knowledge gained from the MOOC. (Survey respondent, Public health researcher, Côte d’Ivoire, Obtained certificate)

## Discussion

The findings reveal that respondents perceived clear benefits from participating in the IR MOOC in relation to the professional activities they subsequently undertook and the results these had for their organisations. Activities included implementing changes to practice, initiating or conducting research, initiating and developing new collaborations, and developing publications and grant applications. Organisational results included benefits to work quality, efficiency or productivity, increased research activities and collaboration, and transfer of knowledge through teaching, training and mentoring.

Findings also reveal, however, that respondents perceived a lack of support for IR practice by supervisors, colleagues and organisations, in terms of funding, support and mentoring. Other commonly cited barriers by respondents include (1) a lack of understanding of IR by colleagues and the wider community, (2) resistance to change by colleagues, supervisors, policy-makers and organisations, (3) the ability to identify suitable collaborators, (4) bureaucracy and administrative and structural barriers. A number of potential solutions to these challenges could be piloted in the future. Running the IR MOOC in collaboration with donors, such as the Global Fund, could increase the availability of financial support for participants as donors increase their emphasis on IR projects. Mentorship programme for IR trainees are under development now, with the aim of increasing technical support to newly trained investigators. These programmes, if successful, should be institutionalised within training initiatives to help ensure the skills developed through the IR MOOC are applied. Organisational readiness for change is crucial for successful implementation of innovations and evidence-based practice in healthcare and human services.^25^ Studies reveal a relationship between a health organisation’s culture and social context and clinicians’ attitudes towards and adoption of evidence-based practice.^26^

Through this study and others,^11 13^ the IR MOOC has been demonstrated to be a valuable tool in communicating IR concepts to learners, engaging a diverse set of participants, and catalysing professional change at the individual and organisational level. Importantly, research is now underway on how to best address barriers around institutional demand for IR and need for professional networks. The next generation of training models can use the IR MOOC as a component of a suite of training tools that strengthen the skills of both individuals and teams in combination with practiced-based learning and guided mentorship.^27^

### Study limitations

A key limitation is that this evaluation is based on the two first offerings of the MOOC and does not include later iterations, including those offered in French or Spanish. Other limitations to this study include response bias, as there may be differences in those who responded to the survey and interview requests compared with those who do not.^28^ Further, as survey and interview respondents are highly self-selective, this may be linked to learners with autonomous motivations for behaviour.^29 30^ Additionally, validity issues may be present, as data are self-reported by participants. A final limitation is the lack of a control group. The authors acknowledge that the results would be strengthened by interviewing employers and organisations to demonstrate the results and impact reported on in this study.

10.1136/bmjopen-2021-054719.supp1 Supplementary data

## Data Availability

Data are available on reasonable request. The datasets used during the current study are available on reasonable request.
