# Supplementary material for: Evaluating results of the implementation research MOOC using Kirkpatrick’s four-level model: a cross-sectional mixed-methods study
Source: BMJ Open. 2022 May 3;12(5):e054719. doi: 10.1136/bmjopen-2021-054719 (PMC9066485; doi:10.1136/bmjopen-2021-054719)
Supplement: Supplementary data [file bmjopen-2021-054719supp001.pdf]

Criteria for describing and evaluating training interventions in healthcare professions – CRe-DEPTH

	Item No	Recommendation	Page No
**Development of training**			
	1	Description of the aim or objectives of the training	3
	2	Description of the underlying theoretical framework	3
	3	Description of the developmental process	3
	4	Description of target population and setting of the training	3
	5	Description of the educational resources	3
**Characteristics of the training**			
	6	Description of the content of the training	3
	7	Description of the format	3
	8	Description of the didactic methods of training	3
	9	Description of tailoring of the training	3
**Characteristics of the providers/trainers**			
	10	Description of the providers of the training	3
**Assessment of the training outcomes**			
	11	Description of the measured outcomes	4
	12	Description of the applied assessment method	4

Ross B, *et al. BMJ Open* 2022; 12:e054719. doi: 10.1136/bmjopen-2021-054719
